# Reconstructive Microsurgery—What a Wonderful Life!

**DOI:** 10.1055/a-2521-2409

**Published:** 2025-03-11

**Authors:** Joon Pio Hong, Geoffrey G. Hallock

**Affiliations:** 1 Department of Plastic and Reconstructive Surgery, Asan Medical Center, University of Ulsan College of Medicine, Seoul, Republic of Korea; 2 Division of Plastic Surgery, St. Luke's Hospital, Sacred Heart Division, Allentown, Pennsylvania, United States

**Figure:**
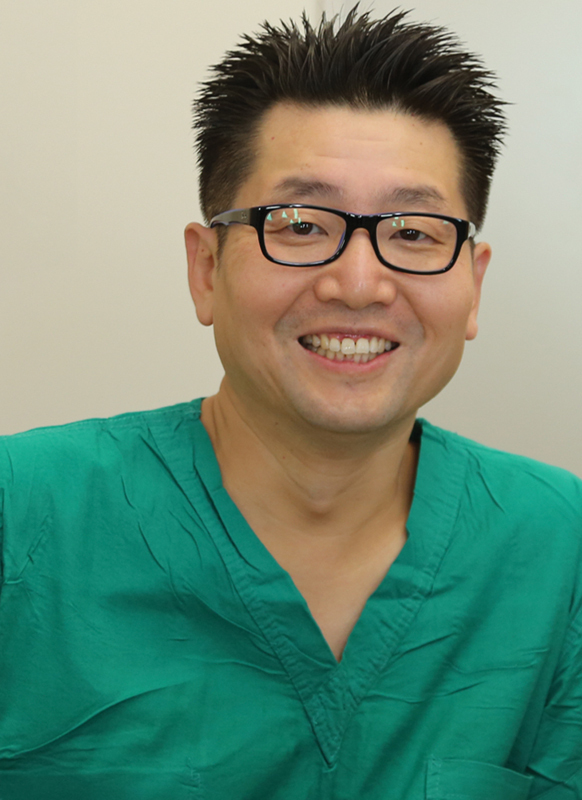
Joon Pio Hong: Editor Emeritus

**Figure:**
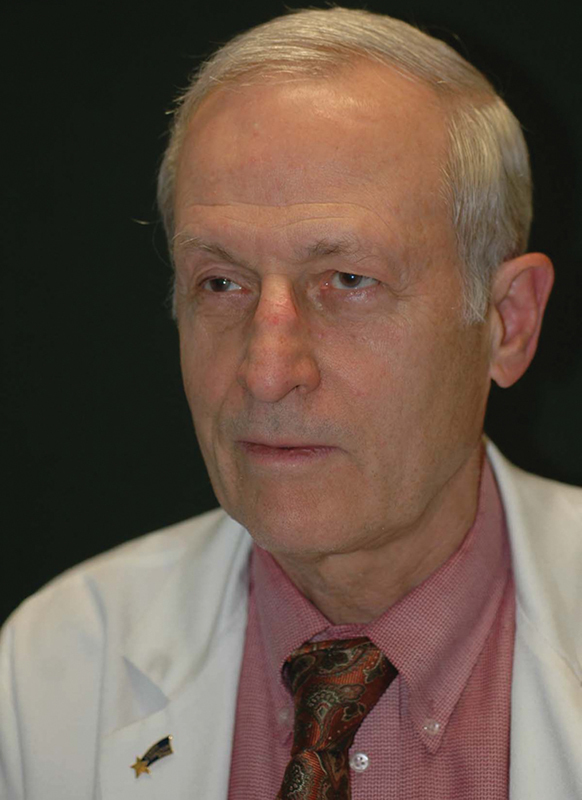
Geoffrey G. Hallock: Associate Editor

“ *I might have had a bad break, but I have an awful lot to live for . . .*

*I consider myself the luckiest man on the face of the earth.* ”

—Lou Gehrig Appreciation Day, July 4, 1939. 1

Woe is us. A day too long. Free flap add-on. Never enough hours. Emergency call awakens us. So too does the “take back.” No wonder no one “likes” us. Always stress and fear of failure. Again. We will be the main event at the next “Morbidity and Mortality” conference. Such destiny requires we meet in the office of the Chief of Surgery once more. Will the malpractice attorney be far behind? And it was all anesthesia's fault, not the “Captain of the Ship.”

So why did we choose this destination? “Doctors” now are just “providers.” 2

Dehumanized. 3 The laws of Goldwyn cannot be revoked—“the dissatisfied patient never moves away.” 4 They just linger in your waiting room. Or “no insurance company ever makes a mistake in your favor.” 4 So must prior authorization be denied, despite our debate with a jury of our peers? Actual payment for services rendered [provided?] is a pittance. Are “free flaps” truly “free” flaps? 5 Every chart will be audited this month to prove our choice of treatment was a “medical necessity” for our “covered lives [sic. patients].” 2 How do we keep the lights on?

Still no time. Working or living on a work/life tightrope. 3 6 Fear the omnipresence of the electronic medical record cash register. Passwords for our passwords. Cell phones with apps so we can quickly text our colleagues—usually while scrubbed. Help us please if we must directly talk to them. Thank goodness the hospital administrators provide “affordable” 2 care by inflicting a new rule for every new day. “Time outs” before “time outs,” yet absolutely conclude that surgery before so does the afternoon. Is anyone interested in what we actually do on the operating table? Don't forget our precious pastime in the laboratory, where that earthshaking project—and our academic rank—the editor just rejected. 7 All this causing BURNOUT. And microsurgeons lead the league. 6

Are we just being selfish? Do our expectations exceed our capabilities? Perhaps a fast-food order clerk, or a bartender for the forlorn would have been a more rewarding profession.

**Fig. 1:**
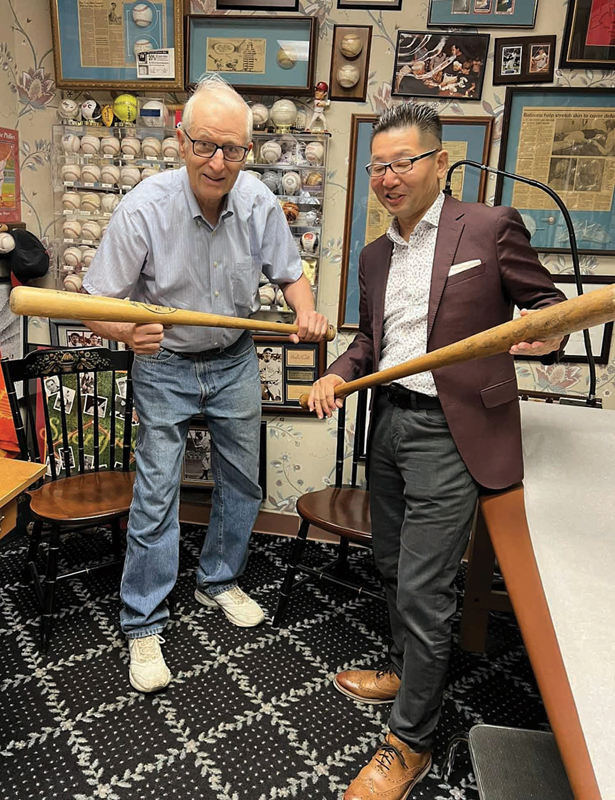
Baseball is a metaphor for our mutual passion. Here in the “cuatro de beísbol,” slugger jp learns to *bunt* , a skill perhaps more difficult than always hitting a home run, yet easily mastered by this nanomicrosurgeon.

Or be a garbage collector [sometimes are we not?]. Better yet digging ditches with a pick or shovel in the sweat of summer or glacial winter. Seek the contentment of a steelworker teetering on the penultimate beam at the top of a skyscraper. Just consider a plumber in the filth of a sewer. Worst yet the baseball “Iron Horse” Lou Gehrig, who had the bad luck to die of a disease for which he is the eponym [amyotrophic lateral sclerosis?]. Did his parting speech at the ballpark whose excerpt quoted above say it right? Gehrig knew he was the luckiest man on Earth because he lived his passion— **Baseball was life** [ Fig. 1 ]!

So too are not we living our passion? Do we just need to awaken and rediscover the **wonder** in our life, just as Jimmy Stewart did almost too late in the classic cinema “It's a Wonderful Life”? 8 Working and living may be difficult to balance, but cannot joy in work exceed that of “life?” 3 6 As Bajaj astutely states, 6 find in work those “opportunities to build relations and networking,” as with our numerous friends worldwide who already share a common interest. Such teamwork always simplifies the task and promotes efficiency. No day ever needs to be repetitive. We are never restricted by arbitrary anatomical boundaries. Diversity is our trademark. Innovation is our intellectual challenge. 9 Today, there are robots 10 so we can all be supermicrosurgeons. Tomorrow artificial or *alternative* intelligence will be within our grasp. 11

There will be no conceivable limit but infinity. Remember, we are the problem solvers for ALL surgical subspecialties. 12 We don't just make life longer, but better for others—no questions asked. We add quality to life, by restoring function 13 always in concert with aesthetics. 14 And that quality we restore in turn rejuvenates the quality of our own life. We become the greatest beneficiary of all. Yes, **we have the wonderful life** !!!
